# Dual-Task Gait Cost and Frontal Lobe Integrity in Poststroke: Results From ONDRI

**DOI:** 10.1093/geroni/igaa057.826

**Published:** 2020-12-16

**Authors:** Frederico Pieruccini-Faria, Paula McLaughlin, Benjamin Cornish, Malcolm Binns, Robert Bartha, Bill McIlroy, ONDRI Collaborators, Manuel Montero Odasso, Frederico Faria

**Affiliations:** 1 University of Western Ontario, London, Ontario, Canada; 2 Queens University, Kingston, Ontario, Canada; 3 University of Waterloo, Waterloo, Ontario, Canada; 4 Baycrest Health Sciences, Toronto, Ontario, Canada; 5 Robarts-Western University, London, Ontario, Canada; 6 Ontario Brain Institute, Toronto, Ontario, Canada; 7 The University of Western Ontario, London, Ontario, Canada

## Abstract

Dual-task gait performance is a marker of motor-cognitive interactions modulated by the frontal lobes. After a stroke, gait disturbances are more evident, particularly when concurrently completing a mental task and walking, an effect called high dual-task cost (DTC). Following a stroke, the potential association of high-DTC, integrity of the frontal lobes and cognitive functioning is unclear. This study screened 161 participants with stroke history from the Ontario Neurodegenerative Disease Research Initiative (ONDRI)-cerebrovascular disease cohort (69.2±7.41 years of age; 31.7% women). Individuals scoring zero in the National Institute of Health Stroke Scale were analyzed (n=102). DTC was the percentage change in gait speed from the single to dual-task condition. Standardized normal-appearing white matter (NAWM) and grey matter (NAGM) volumes from superior, middle and inferior frontal lobe were compared between DTC quartiles (adjusted for age, sex, and education) using a multivariate model (MANOVA), with total frontal lobe volume as a covariate. Another model compared group performance across 5 adjusted cognitive domains (attention, memory, language, visuospatial performance, and executive functioning). Univariate tests revealed that NAWM volume in the superior frontal lobe (F=4.50; p=0.005; partial eta-squared=0.122) was significantly different across DTC quartiles. Contrast tests suggested that the first quartile had larger NAWM than the second and fourth. DTC quartiles also showed differences in attention (F=2.93; p=0.03; partial eta-squared=0.083) and contrast tests indicated that the first quartile performed significantly better than second and fourth. DTC poststroke may be a proxy for structural integrity of superior frontal lobe regions and attention.

